# Cardiovascular health score and its association with postoperative delirium: evidence from the Kailuan study

**DOI:** 10.3389/fmed.2025.1577424

**Published:** 2025-07-09

**Authors:** Zhen-Hua Wang, Yu Jiang, Tao Fang, Jin-Qiu Li, Tai Wang, Chun-Yang Zhou, Rong Wang, Wen-Tao Cai, Hai Liu

**Affiliations:** ^1^Department of Anesthesiology, Kailuan General Hospital, Tangshan, China; ^2^Operating Room, Kailuan General Hospital, Tangshan, China

**Keywords:** postoperative delirium, ideal cardiovascular health score, protective, prospective, surgical patients

## Abstract

**Background:**

Identifying modifiable risk factors for postoperative delirium (POD) is essential for prevention and management. The Ideal Cardiovascular Health Score (CHS), a composite measure of cardiovascular health, has been shown to reduce the risk of various chronic diseases. However, its association with POD has not been extensively explored. This study aims to examine the relationship between CHS and the risk of POD in a cohort of surgical patients.

**Methods:**

Data from the Kailuan Study, a large longitudinal cohort, were used for this analysis. A total of 1,082 participants aged 18–98 years, who underwent non-cardiac surgery under general anesthesia from 2016 to 2021, were included. The CHS was calculated based on seven cardiovascular health metrics: smoking status, body mass index (BMI), physical activity, diet, blood pressure, fasting blood glucose (FBG), and total cholesterol (TC). POD was diagnosed using the Confusion Assessment Method (CAM). Multivariable logistic regression was employed to assess the association between CHS scores and POD, adjusting for potential confounders.

**Results:**

Among the 1,082 participants, 120 developed POD. Higher CHS scores were inversely associated with the risk of POD. Participants with a CHS ≥ 10 had 55% lower odds of developing POD compared to those with a CHS ≤ 7 (OR = 0.45; 95% CI: 0.23–0.89). This protective effect was observed across various subgroups, including age, sex, and alcohol consumption status. Specific CHS components, such as normal blood pressure (OR = 0.49; 95% CI: 0.31–0.78) and FBG < 5.6 mmol/L (OR = 0.65; 95% CI: 0.47–0.94), were independently associated with reduced POD risk.

**Conclusion:**

A higher CHS score is associated with a lower risk of POD, highlighting the potential protective role of cardiovascular health in preventing postoperative complications.

## Introduction

Postoperative delirium (POD) is a common and serious complication following surgery, particularly in elderly patients and those undergoing major procedures. It is characterized by acute disturbances in attention, awareness, and cognition, which can significantly impact postoperative recovery, increasing the length of hospital stays, the risk of functional decline, and the likelihood of mortality (1). The incidence of POD varies widely, with studies indicating that it affects ~10%−30% of surgical patients, and is notably higher in older adults and those undergoing high-risk surgeries, such as cardiac, orthopedic, and neurosurgical procedures (2, 3). Despite its high prevalence and profound implications, POD remains underrecognized and undertreated. A variety of risk factors contribute to its development, including advanced age, preexisting cognitive impairment, comorbid conditions, the type of surgery performed, and perioperative medications (4–6). Identifying these risk factors is critical for early intervention and prevention strategies.

Ideal cardiovascular health score (CHS) is defined by a set of behaviors and health factors that promote optimal cardiovascular function and reduce the risk of cardiovascular disease (7, 8). These include four health behaviors: a healthy diet, regular physical activity, non-smoking status, and optimal weight management; along with three health factors: normal blood pressure, normal cholesterol levels, and normal blood glucose. Research has shown that ideal CHS is not only associated with better cardiovascular outcomes but also with a reduced risk of other chronic conditions, such as diabetes (9), obesity (10), and hypertension (11). Emerging evidence suggests that each component of CHS may independently reduce the incidence of postoperative delirium. For instance, maintaining normal blood pressure (12) and glucose levels (13), as well as adhering to a healthy diet (14), has all been associated with improved cognitive function and a lower risk of delirium after surgery. However, while the individual effects of these CHS metrics on POD have been well documented, the relationship between the overall ideal CHS profile and postoperative delirium remains underexplored.

The aim of this study is to investigate whether a comprehensive approach to cardiovascular health, incorporating all components of CHS, can reduce the incidence of postoperative delirium. Using data from the Kailuan Study, this research will explore the potential protective role of an ideal CHS profile in preventing delirium following surgery.

## Methods

### The Kailuan study and study population

The Kailuan Study is a longitudinal cohort study conducted in the Kailuan community, located in Tangshan, Hebei Province, ~150 km southeast of Beijing. Tangshan is known for its coal mining industry. Between June 2006 and October 2007, the study enrolled 101,510 participants, including 81,110 men and 20,400 women, ranging in age from 18 to 98 years. Participants underwent follow-up medical evaluations every 2 years, with more than 95% of the cohort being of Han Chinese descent (15, 16).

**Figure 1:**
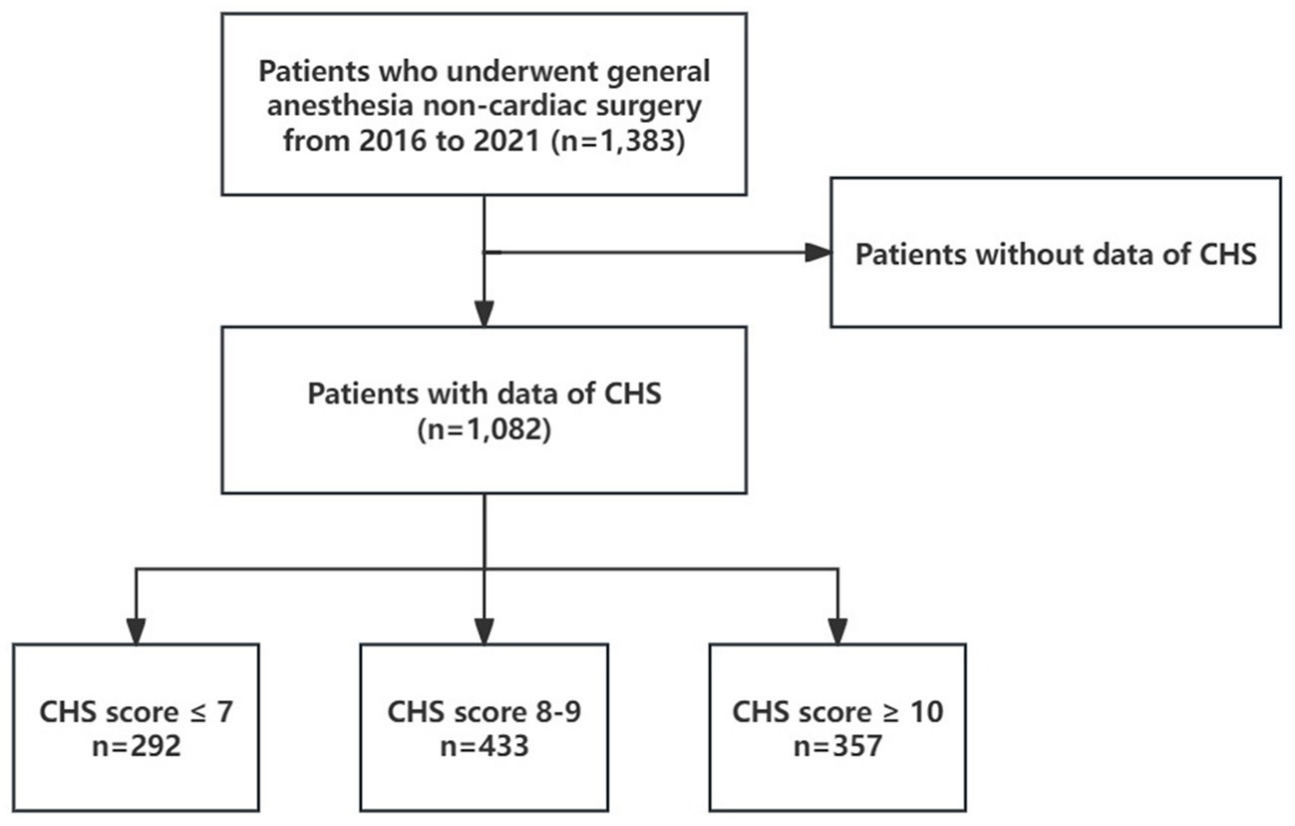
The flowchart of the study population.

This study is derived from the Kailuan Cohort Study as a subset and adopts a prospective cohort design. From 2016 to 2021, a total of 1,383 participants from the Kailuan Study underwent general anesthesia for non-cardiac surgeries. After excluding 301 participants due to missing CHS data, 1,082 participants remained in the final analysis (Figure 1). As this study is part of the Kailuan Cohort Study, individuals with a history of psychiatric disorders or cognitive impairments that precluded them from completing questionnaire assessments were excluded at the time of cohort enrollment. Therefore, all participants included in this analysis were cognitively capable and free from known psychiatric conditions at baseline, ensuring the reliability of self-reported data and reducing potential confounding effects related to mental health status. Ethical approval for this study was obtained from the Ethics Committee of Kailuan General Hospital (Approval No. 2023012; dated March 23, 2023). Written informed consent was obtained from all participants prior to their inclusion in the study.

### Collection of clinical data and relevant definitions

For detailed information on the epidemiological indicators, anthropometric measurements, and biochemical tests used in this study, refer to previously published work by our research team (17, 18). General clinical data for the selected participants were gathered via questionnaire surveys, covering factors such as smoking status, physical activity, salt intake, and histories of diabetes, hypertension, dyslipidemia, heart failure, and medication use. Smoking was defined as consuming at least one cigarette daily on average for the past year, while alcohol consumption was defined as drinking at least 100 mL of hard liquor (≥50% alcohol content) daily for at least 1 year. Participants who had quit smoking or drinking for less than 1 year were still categorized as smokers or drinkers. Regular physical exercise was defined as engaging in physical activity at least three times per week for at least 30 min per session, while occasional exercise was defined as 1–2 times per week, with each session lasting at least 30 min. Anthropometric and biochemical data were extracted from participants' medical examination records. The body mass index (BMI) was calculated as BMI = weight/height^2^ (kg/m^2^).

Blood pressure measurements were taken from the right brachial artery using a calibrated mercury sphygmomanometer. Systolic blood pressure (SBP) and diastolic blood pressure (DBP) were recorded based on the first and fifth Korotkoff sounds, respectively. Three consecutive readings were taken, with a 1–2 min interval between each, and the average value was calculated.

For biochemical testing, fasting blood samples (5 mL) were collected from the median cubital vein after at least 8 h of fasting on the day of the medical examination. A Hitachi 7,600 biochemical analyzer was used to perform standardized tests, including measurements of fasting blood glucose (FBG), total cholesterol (TC), triglycerides (TG), C-reactive protein (CRP), and other relevant indicators.

Intraoperative data, including fluid and blood transfusion volumes, urinary output, blood loss, anesthesia duration, intraoperative blood pressure, surgical complexity, and ASA Physical Status Classification System scores, were obtained from anesthesia records.

In China, surgical procedures—also referred to as operation levels—are officially classified into four levels (Level I to Level IV) according to the “Classification Standards for Surgical Operations” issued by the National Health Commission of the People's Republic of China. This classification is based on factors such as the complexity of the procedure, the potential risk to the patient, the level of surgical skill required, and the need for specialized equipment.

1) Level I: Minor procedures with low risk and technical simplicity, such as superficial skin tumor excision or incision and drainage of abscesses.
2) Level II: Low-to-moderate complexity operations, such as hernia repair or appendectomy.
3) Level III: Moderately complex and higher-risk procedures, including subtotal gastrectomy or partial hepatectomy.
4) Level IV: The most complex and high-risk surgeries, such as liver transplantation, pancreaticoduodenectomy, or open-heart surgery.

This grading system is widely applied in Chinese hospitals to guide clinical management, risk stratification, resource allocation, and surgical performance evaluation.

### Evaluation of health behavior factors

Seven health behavior factors were assessed, with each classified as ideal, moderate, or poor based on specific criteria. The evaluation criteria for each factor were as follows (19):

1) Smoking: No smoking was classified as ideal, past smoking or quitting was moderate, and current smoking was considered poor.
2) BMI: A BMI of <25 kg/m^2^ was considered ideal, 25–29.9 kg/m^2^ was moderate, and ≥30 kg/m^2^ was poor.
3) Physical exercise: Regular exercise (at least three times per week for 30 min) was ideal, occasional exercise (1–2 times per week) was moderate, and no exercise was poor.
4) Healthy diet: Low salt intake (<6 g/day) was considered ideal, moderate salt intake (6–10 g/day) was moderate, and high salt intake (>10 g/day) was poor.

The following health factors were also evaluated:

5) TC: Without medication, <5.18 mmol/L was ideal, 5.18–6.21 mmol/L or <5.18 mmol/L with lipid-lowering medication was moderate, and ≥6.22 mmol/L or ≥5.18 mmol/L with lipid-lowering medication was poor.
6) Blood pressure: Without medication, <120/80 mmHg was ideal, 120–139 and/or 80–89 mmHg or <120/80 mmHg with antihypertensive medication was moderate, and ≥140/90 mmHg or ≥120/80 mmHg with antihypertensive medication was poor.
7) FBG: Without medication, <5.6 mmol/L was ideal, 5.6–6.9 mmol/L or <5.6 mmol/L with antidiabetic medication was moderate, and ≥7.0 mmol/L or ≥5.6 mmol/L with antidiabetic medication was poor.

### CHS calculation and categorization

Each of the seven cardiovascular health behavior factors was assigned a score: two points for an ideal status, one point for a moderate status, and zero points for a poor status. The total score, representing the overall CHS, was calculated by summing the points from all seven factors. Based on the CHS, the population was categorized into three groups: the CHS ≤ 7 group, the 8 ≤ CHS ≤ 9 group, and the CHS ≥ 10 group based on the previous study (20).

### Diagnosis of POD

POD is typically diagnosed based on clinical assessment by trained anesthesiologist, with key diagnostic criteria established by the Confusion Assessment Method (CAM). The Confusion Assessment Method (CAM) is a widely used tool for diagnosing delirium, including POD (21). The CAM assesses four core features: (1) acute onset and fluctuating course, (2) inattention, (3) disorganized thinking, and (4) altered level of consciousness. A diagnosis of delirium is made if both criteria 1 and 2 are present, along with either criteria 3 or 4.

### Statistical analysis

Health examination data were entered by trained staff and uploaded to the computer center at Kailuan General Hospital via a network, using an Oracle 10.2 database. Statistical analysis was performed with SAS software (version 9.4, SAS Institute, Cary, NC, USA). Normally distributed continuous data are expressed as mean ± standard deviation, and group comparisons were made using one-way analysis of variance (ANOVA). For skewed data, results are presented as median (interquartile range), and comparisons were conducted with the Kruskal-Wallis test. Categorical variables are reported as frequencies (percentages), and differences were assessed using the chi-square test. To examine the relationship between continuous CHS and the odds of POD, restricted cubic spline curves were used to model the dose-response association with odds ratios (ORs). Multivariable logistic regression was applied to evaluate the impact of CHS categories on the ORs (95% CI) for POD, and the ORs for each individual CHS factor were analyzed. Subgroup analyses were conducted by age (median split, <75 vs. ≥75 years), sex, and alcohol consumption status. Sensitivity analysis was performed by excluding participants who had undergone brain surgery, followed by logistic regression, to reduce potential bias. A *p-value* of < 0.05 (two-sided) was considered statistically significant.

## Results

### General clinical characteristics

Our study ultimately included 1,082 participants, with 292, 433, and 357 individuals in the CHS score ≤ 7, 8 ≤ CHS score ≤ 9, and CHS score ≥ 10 groups, respectively. Significant differences were observed in age, FBG, BMI, SBP, DBP, TC, CRP, hemoglobin (Hb), and bleeding volume among the three groups (all *p* < 0.05, Table 1). The distribution of men, hypertension, diabetes, chronic heart disease (CHD), stroke, physical activity, salt intake, smoking status, and ICU admission rate also varied significantly between the groups (all *p* < 0.05, Table 1). However, no significant differences were found across the groups in terms of fluid transfusion volume, blood transfusion volume, urinary volume, anesthesia duration, intraoperative hypotension, alcohol consumption status, operation levels, or ASA Physical Status Classification System scores (all *p* > 0.05, Table 1). Additionally, the participants were further categorized based on the presence of POD, and the general clinical data for both groups were analyzed (Supplementary Table 1).

**Table 1:** Baseline characteristics of the study population stratified by CHS scores.

**Variables**	**CHS scores**			***p*-value**
	≤**7**	**8–9**	≥**10**	
*n*	292	433	357	
Age (year)	70.09 ± 4.51	71.64 ± 5.20	71.99 ± 5.74	<0.001
Male gender (%)	275 (994.18)	360 (83.14)	289 (80.95)	<0.001
BMI (Kg/m^2^)	27.05 ± 3.20	25.37 ± 3.21	23.79 ± 2.78	<0.001
SBP (mmHg)	144.90 ± 19.74	138.74 ± 19.09	124.12 ± 15.83	<0.001
DBP (mmHg)	90.89 ± 10.70	86.67 ± 10.07	78.44 ± 9.09	<0.001
TC (mmol/L)	5.54 ± 1.33	5.01 ± 1.09	4.59 ± 1.04	<0.001
FBG (mmol/L)	6.22 ± 1.85	5.35 ± 1.27	4.95 ± 0.81	<0.001
CRP (mg/L)	3.00 (2.40–13.65)	3.00 (1.8–11.20)	3.00 (1.70–11.80)	0.012
Hb (g/L)	139.11 ± 17.81	139.33 ± 17.05	134.92 ± 17.51	<0.001
Fluid transfusion volume (per 100mL)	11.30 (10.00–15.00)	10.00 (10.00–15.00)	10.00 (10.00–15.00)	0.444
Blood transfusion volume (mL)	0.00 (0.00–0.00)	0.00 (0.00–0.00)	0.00 (0.00–0.00)	0.597
Urinary volume (mL)	150 (0.00–200.00)	150 (100.00–200.00)	150 (0.00–200.00)	0.386
Bleeding volume (mL)	20.00 (10.00–50.00)	20.00 (5.00–0.00)	10.00 (5.00–50.00)	0.033
Anesthesia time (h)	2.11 (1.50–3.20)	2.20 (1.50–3.20)	2.06 (1.50–3.00)	0.265
Hypertension (%)	153 (52.40)	204 (47.11)	106 (29.69)	<0.001
Diabetes (%)	90 (30.93)	73 (16.86)	23 (6.46)	<0.001
CHD (%)	61 (20.89)	61 (14.09)	44 (12.32)	0.007
Stroke (%)	65 (22.26)	86 (19.86)	40 (11.20)	<0.001
Intraoperative hypotension (%)	153 (52.40)	221 (51.04)	181 (50.70)	0.903
ICU admission rate (%)	61 (20.89)	56 (12.93)	32 (8.96)	<0.001
**Physical activity (%)**				<0.001
Never	47 (16.10)	30 (6.93)	5 (1.40)	
Occasionally	190 (65.07)	297 (68.59)	225 (63.03)	
Frequently	55 (18.84)	106 (24.48)	127 (35.57)	
**Perceived salt intake (%)**				<0.001
Low (< 6 g/d)	25 (8.56)	40 (9.24)	60 (16.81)	
Intermediate (6–10 g/d)	203 (69.52)	363 (83.83)	286 (80.11)	
High (>10 g/d)	64 (21.92)	30 (6.93)	11 (3.08)	
**Smoking status (%)**				<0.001
Never	84 (28.77)	264 (60.97)	282 (78.99)	
Past or occasionally	35 (11.99)	64 (14.78)	34 (9.52)	
Frequently	173 (59.25)	105 (24.25)	41 (11.48)	
**Drinking status (%)**				0.241
Never	211 (72.26)	325 (75.06)	281 (78.71)	
Past or occasionally	7 (2.40)	12 (2.77)	4 (1.12)	
Frequently	74 (25.34)	96 (22.17)	72 (20.17)	
**Operation levels (%)**				0.422
I (II)	3 (1.03)	4 (0.92)	7 (1.96)	
III	93 (31.85)	112 (25.87)	107 (29.97)	
IV	52 (17.81)	76 (17.75)	64 (17.93)	
Unknown	144 (49.32)	241 (55.66)	179 (50.14)	
**ASA (%)**				0.710
I	2 (0.68)	5 (1.15)	2 (0.56)	
II	200 (68.49)	295 (68.13)	258 (72.27)	
III	82 (28.08)	126 (29.10)	91 (25.49)	
IV(V)	8 (2.74)	7 (1.62)	6 (1.68)	

BMI, Body Mass Index; SBP, Systolic Blood Pressure; DBP, Diastolic Blood Pressure; TC, Total Cholesterol; FBG, Fasting Blood Glucose; CRP, C-Reactive Protein; Hb, Hemoglobin; CHD, Chronic Heart Disease; ASA, American Society of Anesthesiologists; ICU, intensive care unit.

### Relationship between CHS levels and POD

**Figure 2:**
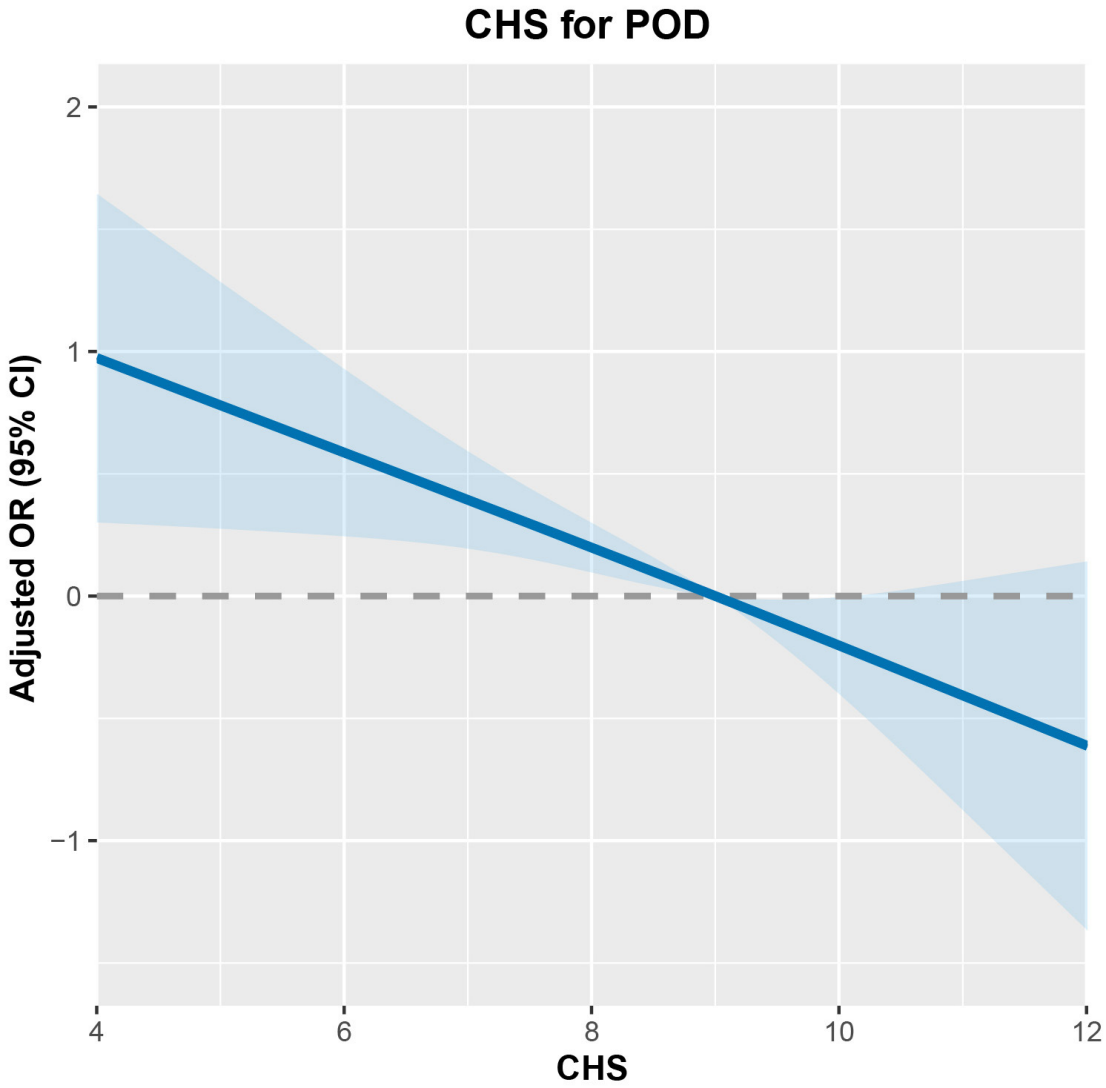
The dose-response relationship between CHS and POD risk.

**Figure 3:**
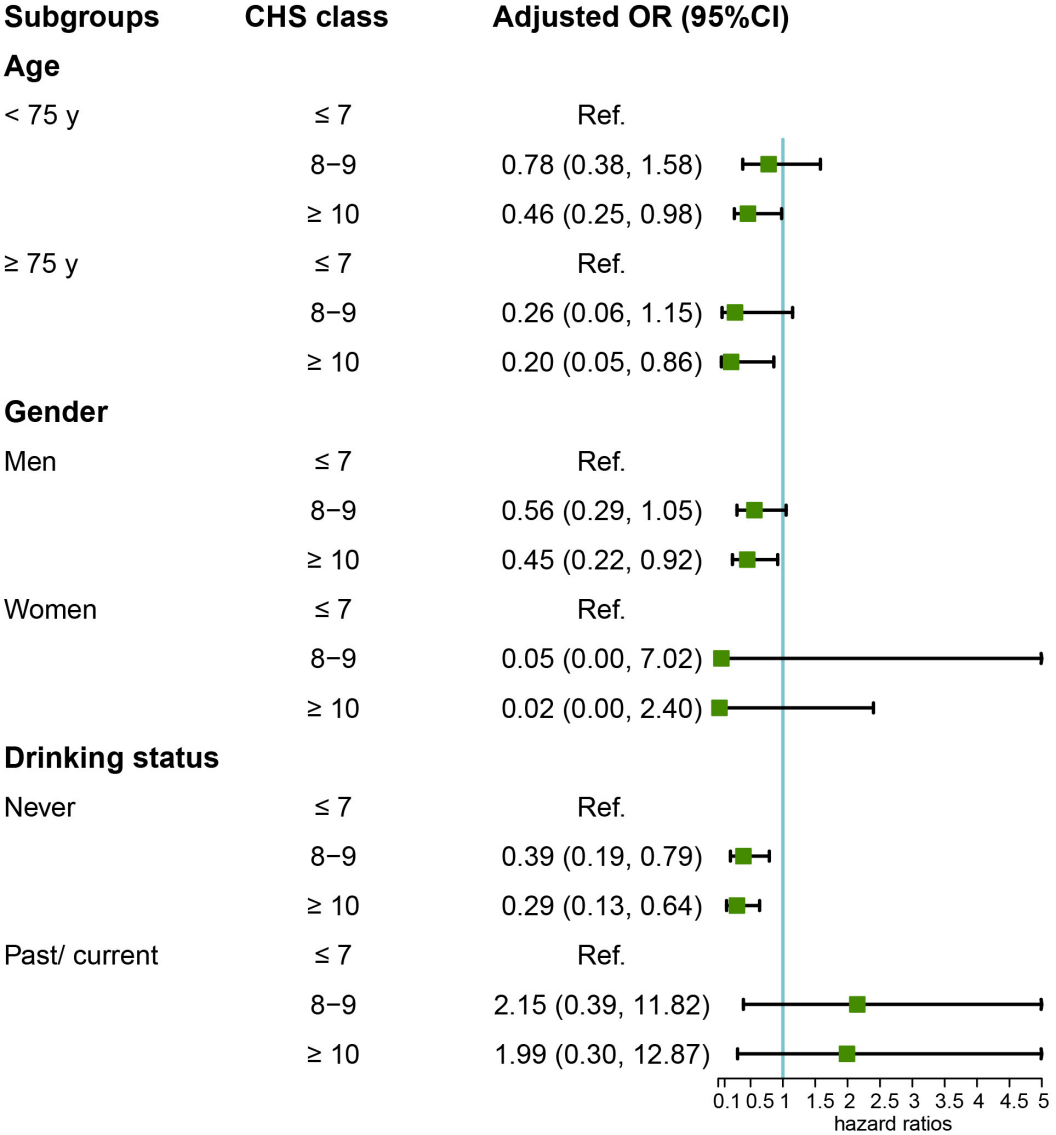
Subgroup analysis of the relationship between CHS and POD risk.

Among the 1,082 participants in the Kailuan Study who underwent general anesthesia for non-cardiac surgery (2016–2021), 120 were diagnosed with POD. CHS scores showed an inverse association with POD risks (Figure 2). After adjusting for confounders, participants with CHS ≥10 had 55% lower odds of POD compared to those with CHS ≤ 7 (OR = 0.45; 95% CI: 0.23–0.89, Table 2). Subgroup analysis confirmed this protective effect in individuals under 75 (OR = 0.46; 95% CI: 0.25–0.98), those 75 or older (OR = 0.20; 95% CI: 0.05–0.86), males (OR = 0.45; 95% CI: 0.22–0.92), and non-drinkers (OR = 0.29; 95% CI: 0.13–0.64, Figure 3).

**Table 2:** The ORs (95%CI) of CHS groups for POD risk.

**CHS**	**AKI cases/*n***	**Crude models**		**Adjusted models**	
		**ORs (95%CI)**	***p*** **-value**	**ORs (95%CI)**	***p*** **-value**
≤7	49/292	Ref.		Ref.	
8–9	46/433	0.59 (0.38, 0.91)	<0.001	0.57 (0.31, 1.07)	0.080
≥ 10	25/357	0.37 (0.22, 0.62)	0.016	0.45 (0.23, 0.89)	0.022
*P* for trend			0.001		0.044

Adjustments were made for age, sex, operation levels, history of stroke and CHD, anesthesia time, drinking status, intraoperative hypotension, CRP, HGB, fluid and blood transfusion volume, urinary volume, bleeding volume, ASA, ICU admission rate, and operation levels.

### CHS components and sensitivity analysis

The association between individual CHS components and POD risk was further examined (Table 3). After adjusting for age, sex, operation level, history of stroke and CHD, anesthesia duration, drinking status, intraoperative hypotension, CRP, Hb, fluid and blood transfusion volume, urinary volume, bleeding volume, ASA, and other CHS components, two key protective factors were identified. Participants with normal blood pressure had a significantly lower risk of POD (OR = 0.49; 95% CI: 0.31–0.78), as did those with FBG < 5.6 mmol/L (OR = 0.65; 95% CI: 0.47–0.94).

**Table 3:** The ORs (95%CI) of each CHS component for POD risk.

**CHS components**	**Crude models**		**Mutually adjusted models**	
	**ORs (95%CI)**	***p*** **-value**	**ORs (95%CI)**	***p*** **-value**
**Smoking status**				
Frequently	Ref.		Ref.	
Past or occasionally	0.82 (0.43, 1.57)	0.548	0.82 (0.42, 1.58)	0.549
Never	0.82 (0.54, 1.24)	0.341	0.74 (0.48, 1.15)	0.178
**BMI (kg/m** ^2^ **)**				
≥30	Ref.		Ref.	
25–29.9	0.72 (0.38, 1.39)	0.326	0.73 (0.37, 1.42)	0.351
<25	0.49 (0.25, 0.95)	0.033	0.55 (0.27, 1.09)	0.087
**Physical exercise**				
Never	Ref.		Ref.	
Occasionally	1.13 (0.55, 2.34)	0.744	1.25 (0.59, 2.64)	0.564
Frequently	0.74 (0.33, 1.66)	0.460	0.77 (0.34, 1.74)	0.524
**Salt intake (g/d)**				
High (>10)	Ref.		Ref.	
Intermediate (6–10)	0.91 (0.49, 1.69)	0.763	0.96 (0.50,1.83)	0.893
**Low (<6)**	0.62 (0.26, 1.47)	0.273	0.60 (0.25, 1.46)	0.257
**TC (mmol/L)**				
≥6.22	Ref.		Ref.	
5.18–6.21	0.60 (0.33, 1.09)	0.095	0.69 (0.37, 1.28)	0.242
<5.18	0.61(0.35, 1.05)	0.073	0.76 (0.43, 1.33)	0.331
**Blood pressure (mmHg)**				
≥140/90	Ref.		Ref.	
120–139/80–89	0.66 (0.36, 1.20)	0.174	0.77 (0.41, 1.44)	0.410
<120/80	**0.46 (0.30, 0.72)**	**<0.001**	**0.49 (0.31, 0.78)**	0.003
**FBG (mmol/L)**				
≥7	Ref.		Ref.	
5.6–6.9	0.73 (0.38, 1.40)	0.342	0.76 (0.39, 1.49)	0.426
<5.6	**0.56 (0.31, 0.98)**	**0.044**	**0.65 (0.47, 0.94)**	**0.012**

Adjustments were made for age, sex, operation levels, history of stroke and CHD, anesthesia time, drinking status, intraoperative hypotension, CRP, HGB, fluid and blood transfusion volume, urinary volume, bleeding volume, ASA, ICU admission rate, operation levels, and all CHS components. Results presented with bold values were statistically significant.

A sensitivity analysis was conducted to address the potential influence of brain surgery on POD risk. Participants who had undergone brain surgery were excluded, and the primary analysis was repeated. The results remained consistent with the main findings, supporting the robustness of the conclusions (Table 4).

**Table 4:** The ORs (95%CI) of CHS groups for POD risk after excluding participants with brain surgery.

**CHS**	**POD cases/*n***	**Crude models**		**Adjusted models**	
		**ORs (95%CI)**	***p*** **-value**	**ORs (95%CI)**	***p*** **-value**
≤7	28/265	Ref.		Ref.	
8–9	29/406	0.63 (0.42, 1.19)	0.239	0.55 (0.24, 1.20)	0.121
≥10	13/331	0.35 (0.18, 0.68)	0.002	0.41 (0.20, 0.81)	0.012
*P* for trend			0.022		0.023

Adjustments were made for age, sex, operation levels, history of stroke and CHD, anesthesia time, drinking status, intraoperative hypotension, CRP, HGB, fluid and blood transfusion volume, urinary volume, bleeding volume, ASA, ICU admission rate, and operation levels.

## Discussion

Identifying modifiable risk factors is essential for the prevention and management of POD. This study revealed a strong association between CHS scores and POD risk, with higher CHS scores linked to a reduced likelihood of POD, particularly among men and non-drinkers. Additionally, specific CHS components, such as normal blood pressure and normal fasting blood glucose, were independently associated with a lower risk of POD. These findings offer both theoretical support and clinical evidence for strategies aimed at reducing POD risk.

The CHS score is a comprehensive measure of cardiovascular health, incorporating several modifiable risk factors, including smoking status, BMI, physical activity, diet, blood pressure, blood glucose, and cholesterol levels. While the relationship between CHS and POD has been less explored, previous studies have demonstrated that higher CHS scores are linked to a reduced risk of various adverse health outcomes. These include lower incidence rates of cardiovascular diseases (such as coronary artery disease, stroke, and heart failure) (22–24), reduced all-cause mortality (25), improved metabolic health (including obesity, type 2 diabetes, and metabolic syndrome) (22, 24), and better cognitive function (26). In 2010, the American Heart Association (AHA) set a goal to improve population cardiovascular health by 20% and reduce mortality from cardiovascular diseases and stroke by 20% by 2020 (27). Achieving these targets requires the implementation of innovative strategies for promoting cardiovascular health and preventing disease, leveraging AHA's research, clinical insights, public health policies, and advocacy initiatives for long-term impact.

Although research on the association between overall CHS and POD is limited, the relationship between individual CHS components and POD has been extensively studied. For instance, blood pressure control has been shown to play a crucial role in reducing POD risk, as both hypertension and intraoperative hypotension have been linked to cerebral hypoperfusion and cognitive impairment (28, 29). Fasting blood glucose is another key factor, as hyperglycemia and glycemic fluctuations during the perioperative period have been associated with a higher risk of POD due to increased oxidative stress and neuroinflammation (30, 31). Physical activity has been reported to improve cognitive reserve, which may protect against cognitive decline, including POD (32). Similarly, BMI is linked to metabolic and vascular health, with obesity being a known risk factor for cognitive dysfunction (33), while smoking is associated with neurovascular injury and oxidative stress, both of which increase POD risk (34). Cholesterol levels also play a role, as dyslipidemia contributes to atherosclerosis and cerebrovascular dysfunction, which are implicated in the pathophysiology of POD (14). Lastly, dietary habits, particularly the consumption of high-sodium or nutrient-deficient diets, have been linked to cognitive impairment and increased POD susceptibility (35). Collectively, these findings underscore the potential for holistic cardiovascular health promotion to reduce the risk of POD.

The association between higher CHS scores and lower POD risk appears to be more evident in non-drinkers than in drinkers, possibly due to the neurotoxic effects of alcohol, metabolic disturbances, and impaired liver function. Chronic alcohol consumption induces structural and functional changes in the brain, including cortical atrophy, white matter damage, and disruptions in neurotransmitter systems such as γ-aminobutyric acid (GABA) and glutamate, thereby reducing cognitive reserve and heightening susceptibility to POD (36–38). Additionally, alcohol use is associated with systemic inflammation, oxidative stress, and metabolic dysregulation, including impaired glucose metabolism and dyslipidemia, which may counteract the protective effects of optimal CHS components, such as normal blood glucose and cholesterol levels (39, 40). Liver dysfunction, a common consequence of chronic alcohol consumption, can impair the clearance of anesthetic agents, leading to prolonged neurotoxic exposure during surgery, further increasing POD risk (41). Moreover, drinkers may exhibit poorer adherence to other healthy lifestyle behaviors, such as regular physical activity, balanced nutrition, and blood pressure control, all of which are integral components of CHS. These factors may collectively weaken the protective influence of CHS on POD in drinkers, highlighting the need for targeted preventive strategies that consider alcohol consumption as a potential modifier of POD risk.

The potential mechanisms underlying the association between CHS and POD are multifactorial, involving pathways related to cerebral perfusion, neuroinflammation, oxidative stress, and metabolic health. Optimal cardiovascular health promotes stable cerebral blood flow, reducing the likelihood of cerebral hypoperfusion, a key contributor to POD (42). Components such as blood pressure regulation are critical, as both hypertension and intraoperative hypotension can impair cerebral autoregulation, leading to ischemic injury and cognitive dysfunction (43). Glycemic control also plays a pivotal role, as hyperglycemia and glycemic variability increase oxidative stress (44), promote neuroinflammation (45), and disrupt the blood-brain barrier, all of which heighten the risk of delirium. Cholesterol homeostasis is essential for maintaining neuronal membrane integrity and synaptic plasticity, while dyslipidemia has been linked to cognitive decline through its association with cerebrovascular disease (46). Lifestyle factors such as physical activity, non-smoking status, and healthy dietary habits support neuroplasticity, reduce systemic inflammation, and enhance overall cognitive reserve, thereby mitigating POD risk (47–49). Collectively, these mechanisms suggest that achieving optimal CHS may provide neuroprotective effects during the perioperative period, reducing brain vulnerability to anesthesia, surgical stress, and systemic inflammatory responses. This highlights the potential for cardiovascular health optimization as a strategy to prevent POD.

This study provides a novel exploration of the relationship between CHS and postoperative delirium (POD), offering insights into potential strategies for perioperative risk assessment and management. Its strengths include a large sample size, comprehensive adjustment for relevant covariates, and the application of multiple statistical approaches. Nonetheless, several limitations should be noted. First, the Kailuan community, composed primarily of industrial workers, may introduce sex imbalance; this was addressed through sex-stratified analyses to minimize potential bias. Second, CHS was measured only once, which may not reflect temporal fluctuations, potentially leading to misclassification. Future studies should consider longitudinal assessments to better capture dynamic changes in health status. Third, data on specific surgical types were unavailable, limiting the evaluation of procedure-specific risks for POD. To account for surgical complexity, we incorporated operation level (surgical grade)—a standardized classification in China reflecting surgical difficulty and risk—as a covariate and stratification factor. Although this serves as a reasonable proxy for invasiveness, future research should collect detailed information on surgical types and indications. Furthermore, postoperative analgesia protocols vary across institutions in China, and specific analgesic data were not available in this study. Since analgesia intensity is generally correlated with surgical complexity, operation level may also partially reflect postoperative pain management. Lastly, data on medications known to influence POD risk—such as anticholinergics, antidepressants, opioids, and benzodiazepines—were not collected. However, as part of the Kailuan cohort, individuals with psychiatric disorders were excluded at baseline, likely reducing psychotropic medication use in the study population. Still, the potential influence of unmeasured drug exposures cannot be entirely ruled out.

## Conclusions

The findings of this study indicate a strong association between the CHS and the risk of POD, with higher CHS scores linked to a reduced risk of POD, particularly among men. Promoting and maintaining optimal cardiovascular health, as reflected by the CHS, should be prioritized in public health initiatives. Efforts to enhance cardiovascular health should focus on lifestyle changes, effective risk factor management, and increasing awareness of the key metrics that define the CHS.

## Data Availability

The raw data supporting the conclusions of this article will be made available by the authors, without undue reservation.

## References

[1] Swarbrick CJ Partridge JSL. Evidence-based strategies to reduce the incidence of postoperative delirium: a narrative review. Anaesthesia. (2022) 77:92–101. 10.1111/anae.15607 35001376

[2] Yan E Veitch M Saripella A Alhamdah Y Butris N Tang-Wai DF. Association between postoperative delirium and adverse outcomes in older surgical patients: a systematic review and meta-analysis. J Clin Anesth. (2023) 90:111221. 10.1016/j.jclinane.2023.111221 37515876

[3] Xiao MZ Liu CX Zhou LG Yang Y Wang Y. Postoperative delirium, neuroinflammation, and influencing factors of postoperative delirium: a review. Medicine. (2023) 102:e32991. 10.1097/MD.0000000000032991 36827061 PMC11309669

[4] Aldecoa C Bettelli G Bilotta F Sanders RD Aceto P Audisio R. Update of the European society of anaesthesiology and intensive care medicine evidence-based and consensus-based guideline on postoperative delirium in adult patients. Eur J Anaesthesiol. (2024) 41:81–108. 10.1097/EJA.0000000000001876 37599617 PMC10763721

[5] Viderman D Brotfain E Bilotta F Zhumadilov A. Risk factors and mechanisms of postoperative delirium after intracranial neurosurgical procedures. Asian J Anesthesiol. (2020) 58:5–13. 10.6859/aja.202003_58(1).0002 33081429

[6] Mevorach L Forookhi A Farcomeni A Romagnoli S Bilotta F. Perioperative risk factors associated with increased incidence of postoperative delirium: systematic review, meta-analysis, and grading of recommendations assessment, development, and evaluation system report of clinical literature. Br J Anaesth. (2023) 130:e254–62. 10.1016/j.bja.2022.05.032 35810005

[7] Michos ED Khan SS. Further understanding of ideal cardiovascular health score metrics and cardiovascular disease. Expert Rev Cardiovasc Ther. (2021) 19:607–17. 10.1080/14779072.2021.1937127 34053373 PMC8338780

[8] Te Hoonte F Spronk M Sun Q Wu K Fan S Wang Z. Ideal cardiovascular health and cardiovascular-related events: a systematic review and meta-analysis. Eur J Prev Cardiol. (2024) 31:966–85. 10.1093/eurjpc/zwad405 38149986

[9] Sun Y Yu Y Zhang K Yu B Yu Y Wang Y. Association between Life's Essential 8 score and risk of premature mortality in people with and without type 2 diabetes: a prospective cohort study. Diabetes Metab Res Rev. (2023) 39:e3636. 10.1002/dmrr.3636 36918526

[10] Broni EK Perdigao JL Koelper N Lewey J Levine LD. Ideal cardiovascular health and adipokine levels: the multi-ethnic study of atherosclerosis. Endocr Pract. (2023) 29:456–64. 10.1016/j.eprac.2023.03.276 37028649 PMC10330128

[11] Zhao HY Liu XX Wang AW Wu YT Zheng XM Zhao XH. Ideal cardiovascular health and incident hypertension: the longitudinal community-based Kailuan study. Medicine. (2016) 95:e5415. 10.1097/MD.0000000000005415 27977580 PMC5268026

[12] Jin ZJ. Hu, Ma D. Postoperative delirium: perioperative assessment, risk reduction, and management. Br J Anaesth. (2020) 125:492–504. 10.1016/j.bja.2020.06.063 32798069

[13] Liu S Xv L Wu X Wang F Wang J Tang X. Potential value of preoperative fasting blood glucose levels in the identification of postoperative delirium in non-diabetic older patients undergoing total hip replacement: the perioperative neurocognitive disorder and biomarker lifestyle study. Front Psychiatry. (2022) 13:941048. 10.3389/fpsyt.2022.941048 36311514 PMC9606582

[14] Zhao Y Zhong K Zheng Y Xia X Lin X Kowark A. Postoperative delirium risk in patients with hyperlipidemia: a prospective cohort study. J Clin Anesth. (2024) 98:111573. 10.1016/j.jclinane.2024.111573 39094442

[15] Liu T Wang Y Wang X Liu C Zhang Q Song M. Habitually skipping breakfast is associated with the risk of gastrointestinal cancers: evidence from the kailuan cohort study. J Gen Intern Med. (2023) 38:2527–36. 10.1007/s11606-023-08094-7 36869181 PMC10465444

[16] Liu C Zhang Q Liu T Zhang Q Song M Ruan G. Predicted lean body mass trajectories, and cancer risk and cancer-specific and all-cause mortality: a prospective cohort study. J Cachexia Sarcopenia Muscle. (2023) 14:2916–24. 10.1002/jcsm.13370 37969022 PMC10751432

[17] Liu T Liu CA Zhang QS Zhang Q Wang YM Song MM. Association of the age of onset of metabolic syndrome with the risk of all cancer types. Diabetes Metab Syndr. (2023) 17:102896. 10.1016/j.dsx.2023.102896 37913630

[18] Deng L Liu T Liu CA Zhang Q Song MM Lin SQ. The association of metabolic syndrome score trajectory patterns with risk of all cancer types. Cancer. (2024) 130:2150–9. 10.1002/cncr.35235 38462898

[19] Xue H Wang J Hou J Gao J Chen S Zhu H. Ideal cardiovascular health behaviors and factors and high sensitivity C-reactive protein: the Kailuan cross-sectional study in Chinese. Clin Chem Lab Med. (2014) 52:1379–86. 10.1515/cclm-2013-0657 24791822

[20] Zhou H Ding X Wu S Yan J Cao J. Association of cardiovascular health score trajectory and risk of subsequent cardiovascular disease in non-diabetic population: a cohort study. BMC Public Health. (2023) 23:1043. 10.1186/s12889-023-15569-z 37264382 PMC10233877

[21] Inouye SK van Dyck CH Alessi CA Balkin S Siegal AP Horwitz RI. Clarifying confusion: the confusion assessment method. A new method for detection of delirium. Ann Intern Med. (1990) 113:941–8. 10.7326/0003-4819-113-12-941 2240918

[22] Douglas PS McCallum S Lu MT Umbleja T Fitch KV Foldyna B. Ideal cardiovascular health, biomarkers, and coronary artery disease in persons with HIV. Aids. (2023) 37:423–34. 10.1097/QAD.0000000000003418 36525544 PMC9877147

[23] Ying Y Lin S Kong F Li Y Xu S Liang X. Ideal cardiovascular health metrics and incidence of ischemic stroke among hypertensive patients: a prospective cohort study. Front Cardiovasc Med. (2020) 7:590809. 10.3389/fcvm.2020.590809 33330652 PMC7719670

[24] Li Y Gray A Xue L Farb MG Ayalon N Andersson C. Metabolomic profiles, ideal cardiovascular health, and risk of heart failure and atrial fibrillation: insights from the framingham heart study. J Am Heart Assoc. (2023) 12:e028022. 10.1161/JAHA.122.028022 37301766 PMC10356055

[25] Zhang J Chen G Habudele Z Wang X Cai M Li H. Relation of Life's Essential 8 to the genetic predisposition for cardiovascular outcomes and all-cause mortality: results from a national prospective cohort. Eur J Prev Cardiol. (2023) 30:1676–85. 10.1093/eurjpc/zwad179 37228091

[26] Shen R Guo X Zou T Ma L. Association of cardiovascular health with cognitive function in US older adults: a population-based cross-sectional study. Dement Geriatr Cogn Disord. (2024) 53:1–11. 10.1159/000534923 37980885

[27] Lloyd-Jones DM Hong Y Labarthe D Mozaffarian D Appel LJ Van Horn L. Defining and setting national goals for cardiovascular health promotion and disease reduction: the American Heart Association's strategic impact goal through 2020 and beyond. Circulation. (2010) 121:586–613. 10.1161/CIRCULATIONAHA.109.192703 20089546

[28] Qureshi O Arthur ME. Recent advances in predicting, preventing, and managing postoperative delirium. Fac Rev. (2023) 12:19. 10.12703/r/12-19 37529149 PMC10388843

[29] D'Amico F Fominskiy EV Turi S Pruna A Fresilli S Triulzi M. Intraoperative hypotension and postoperative outcomes: a meta-analysis of randomised trials. Br J Anaesth. (2023) 131:823–31. 10.1016/j.bja.2023.08.026 37739903

[30] Park SJ Oh AR Lee JH Yang K Park J. Association of preoperative blood glucose level with delirium after non-cardiac surgery in diabetic patients. Korean J Anesthesiol. (2024) 77:226–35. 10.4097/kja.23301 38171594 PMC10982528

[31] Liu K Song Y Yuan Y Li Z Wang X Zhang W. Type 2 diabetes mellitus with tight glucose control and poor pre-injury stair climbing capacity may predict postoperative delirium: a secondary analysis. Brain Sci. (2022) 12:951. 10.3390/brainsci12070951 35884759 PMC9317912

[32] Lee SS Lo Y Verghese J. Physical activity and risk of postoperative delirium. J Am Geriatr Soc. (2019) 67:2260–6. 10.1111/jgs.16083 31368511 PMC6861610

[33] Feinkohl I Janke J Slooter AJC Winterer G Spies C Pischon T. Metabolic syndrome and the risk of postoperative delirium and postoperative cognitive dysfunction: a multi-centre cohort study. Br J Anaesth. (2023) 131:338–47. 10.1016/j.bja.2023.04.031 37344340

[34] Zhou S Shi S Xie C Chen G. Association between smoking and postoperative delirium in surgical patients with pulmonary hypertension: a secondary analysis of a cohort study. BMC Psychiatry. (2022) 22:371. 10.1186/s12888-022-03981-5 35650551 PMC9158079

[35] Kou C Zhao X Fan X Lin X Wang Q Yu J. Dietary sodium/potassium intake and cognitive impairment in older patients with hypertension: data from NHANES 2011-2014. J Clin Hypertens. (2023) 25:534–44. 10.1111/jch.14667 37183770 PMC10246459

[36] Lees B Meredith LR Kirkland AE Bryant BE. Squeglia LM. Effect of alcohol use on the adolescent brain and behavior. Pharmacol Biochem Behav. (2020) 192:172906. 10.1016/j.pbb.2020.172906 32179028 PMC7183385

[37] Nippert KE Rowland CP Vazey EM Moorman DE. Alcohol, flexible behavior, and the prefrontal cortex: functional changes underlying impaired cognitive flexibility. Neuropharmacology. (2024) 260:110114. 10.1016/j.neuropharm.2024.110114 39134298 PMC11694314

[38] Field M Schoenmakers T Wiers RW. Cognitive processes in alcohol binges: a review and research agenda. Curr Drug Abuse Rev. (2008) 1:263–79. 10.2174/1874473710801030263 19630725 PMC3066447

[39] Bishehsari F Magno E Swanson G Desai V Voigt RM Forsyth CB. Alcohol and gut-derived inflammation. Alcohol Res. (2017) 38:163–71. 28988571 10.35946/arcr.v38.2.02 PMC5513683

[40] Avogaro A Tiengo A. Alcohol, glucose metabolism and diabetes. Diabetes Metab Rev. (1993) 9:129–46. 10.1002/dmr.5610090205 8258307

[41] Mackowiak B Fu Y Maccioni L Gao B. Alcohol-associated liver disease. J Clin Invest. (2024) 134:e176345. 10.1172/JCI176345 38299591 PMC10836812

[42] Ryman SG Vakhtin AA Mayer AR van der Horn HJ Shaff NA Nitschke SR. Abnormal cerebrovascular activity, perfusion, and glymphatic clearance in lewy body diseases. Mov Disord. (2024) 39:1258–68. 10.1002/mds.29867 38817039 PMC11341260

[43] Reed G Devous M. Cerebral blood flow autoregulation and hypertension. Am J Med Sci. (1985) 289:37–44. 10.1097/00000441-198501000-00007 3881953

[44] Franco C Sciatti E Favero G Bonomini F Vizzardi E Rezzani R. Essential hypertension and oxidative stress: novel future perspectives. Int J Mol Sci. (2022) 23:14489. 10.3390/ijms232214489 36430967 PMC9692622

[45] Santisteban MM Ladecola C Carnevale D. Hypertension, neurovascular dysfunction, and cognitive impairment. Hypertension. (2023) 80:22–34. 10.1161/HYPERTENSIONAHA.122.18085 36129176 PMC9742151

[46] Kivipelto M Mangialasche F Ngandu T. Lifestyle interventions to prevent cognitive impairment, dementia and Alzheimer disease. Nat Rev Neurol. (2018) 14:653–66. 10.1038/s41582-018-0070-3 30291317

[47] Cassilhas RC Tufik S de Mello MT. Physical exercise, neuroplasticity, spatial learning and memory. Cell Mol Life Sci. (2016) 73:975–83. 10.1007/s00018-015-2102-0 26646070 PMC11108521

[48] Pandria N Athanasiou A Styliadis C Terzopoulos N Mitsopoulos K Paraskevopoulos E. Does combined training of biofeedback and neurofeedback affect smoking status, behavior, and longitudinal brain plasticity? Front Behav Neurosci. (2023) 17:1096122. 10.3389/fnbeh.2023.1096122 36778131 PMC9911884

[49] Rajaram S Jones J Lee GJ. Plant-based dietary patterns, plant foods, and age-related cognitive decline. Adv Nutr. (2019) 10:S422–36. 10.1093/advances/nmz081 31728502 PMC6855948

