# Supplementary material for: Cardiovascular health score and its association with postoperative delirium: evidence from the Kailuan study
Source: Front Med (Lausanne). 2025 Jul 9;12:1577424. doi: 10.3389/fmed.2025.1577424 (PMC12283702; doi:10.3389/fmed.2025.1577424)
Supplement: Supplementary file 1 [file Table_1.DOCX]

**Tables**

**Supplementary Table 1. Baseline characteristics of the study population stratified by POD status.**

|  | **Non-POD** | **POD** | **p-value** |
| --- | --- | --- | --- |
| **n** | 962 | 120 |  |
| **Age (year)** | 71.26±5.25 | 71.97±5.35 | <0.001 |
| **Male gender (%)** | 819(85.14) | 105(87.50) | 0.489 |
| **BMI (Kg/m^2^)** | 25.22±3.32 | 25.92±3.27 | <0.001 |
| **SBP (mmHg)** | 134.91±19.79 | 140.97±21.76 | <0.001 |
| **DBP (mmHg)** | 84.75±10.94 | 88.20±11.99 | <0.001 |
| **TC (mmol/L)** | 5.01±1.14 | 5.08±1.58 | <0.001 |
| **FBG (mmol/L)** | 5.42±1.41 | 5.67±1.54 | <0.001 |
| **HGB (g/L)** | 138.16±17.20 | 135.08±19.71 | 0.001 |
| **CRP (mg/L)** | 3.00(1.80-11.60) | 4.20(3.00-16.85) | 0.003 |
| **Fluid transfusion volume (per 100mL)** | 10.00(10.00-15.00) | 15.00(10.00-20.00) | <0.001 |
| **Blood transfusion volume (mL)** | 0.00(0.00-0.00) | 0.00(0.00-100.00) | 0.022 |
| **Urinary volume (mL)** | 150(0-200) | 150(10 -300) | 0.009 |
| **Bleeding volume (mL)** | 10.00(5.00-50.00) | 50.00(10.00-200.00) | <0.001 |
| **Anesthesia time (h)** | 2.10(1.50-3.10) | 2.85(1.84-3.75) | <0.001 |
| **Hypertension (%)** | 402(41.79) | 61(50.83) | 0.059 |
| **Diabetes (%)** | 160(16.67) | 26(21.67) | 0.171 |
| **CHD (%)** | 143(14.86) | 23(19.17) | 0.218 |
| **Stroke (%)** | 166(17.26) | 25(20.83) | 0.332 |
| **Intraoperative hypotension (%)** | 482(50.10) | 73(60.83) | 0.027 |
| **Physical activity (%)** |  |  | 0.208 |
| **Never** | 73(7.59) | 9(7.50) |  |
| **Occasionally** | 625(64.97) | 87(72.50) |  |
| **Frequently** | 264(27.44) | 24(20.00) |  |
| **Perceived salt intake (%)** |  |  | 0.481 |
| **Low (<6 g/d)** | 92(9.56) | 13(10.83) |  |
| **Intermediate (6-10 g/d)** | 755(78.48) | 97(11.38) |  |
| **High (>10 g/d)** | 115(11.95) | 10(8.33) |  |
| **Smoking status (%)** |  |  | 0.618 |
| **Never** | 279(29.00) | 40(33.33) |  |
| **Past or occasionally** | 119(12.37) | 14(11.67) |  |
| **Frequently** | 564(58.63) | 66(55.00) |  |
| **Drinking status (%)** |  |  | 0.279 |
| **Never** | 720(74.84) | 97(80.83) |  |
| **Past or occasionally** | 22(2.29) | 1(0.83) |  |
| **Frequently** | 220(22.87) | 22(18.33) |  |
| **Operation levels (%)** |  |  | 0.053 |
| **Ⅰ (Ⅱ)** | 13(1.35) | 1(0.83) |  |
| **Ⅲ** | 279(29.00) | 33(27.05) |  |
| **Ⅳ** | 160(16.63) | 32(26.67) |  |
| **Unknown** | 510(53.01) | 54(45.00) |  |
| **ASA (%)** |  |  | 0.001 |
| **Ⅰ** | 8(0.83) | 1(0.83) |  |
| **Ⅱ** | 680(70.69) | 73(60.83) |  |
| **Ⅲ** | 261(27.13) | 38(31.67) |  |
| **Ⅳ(Ⅴ)** | 13(1.35) | 8(6.67) |  |

**Note: BMI, body mass index; SBP, systolic blood pressure; DBP, diastolic blood pressure; TC, total cholesterol; FBG: fasting blood glucose; CRP: C-reactive protein; Hb, hemoglobin; CHD: chronic heart disease; ASA: ASA physical status classification system.**
